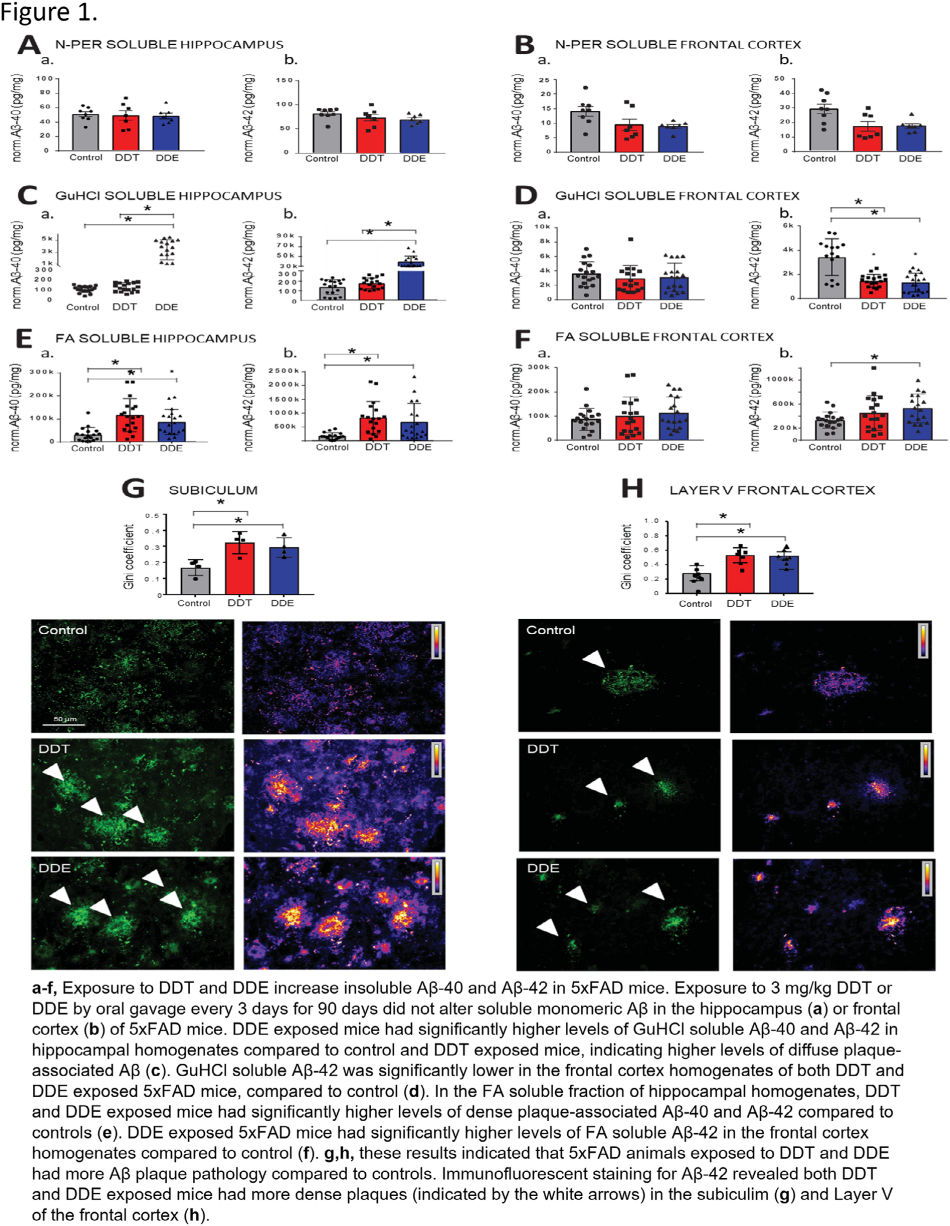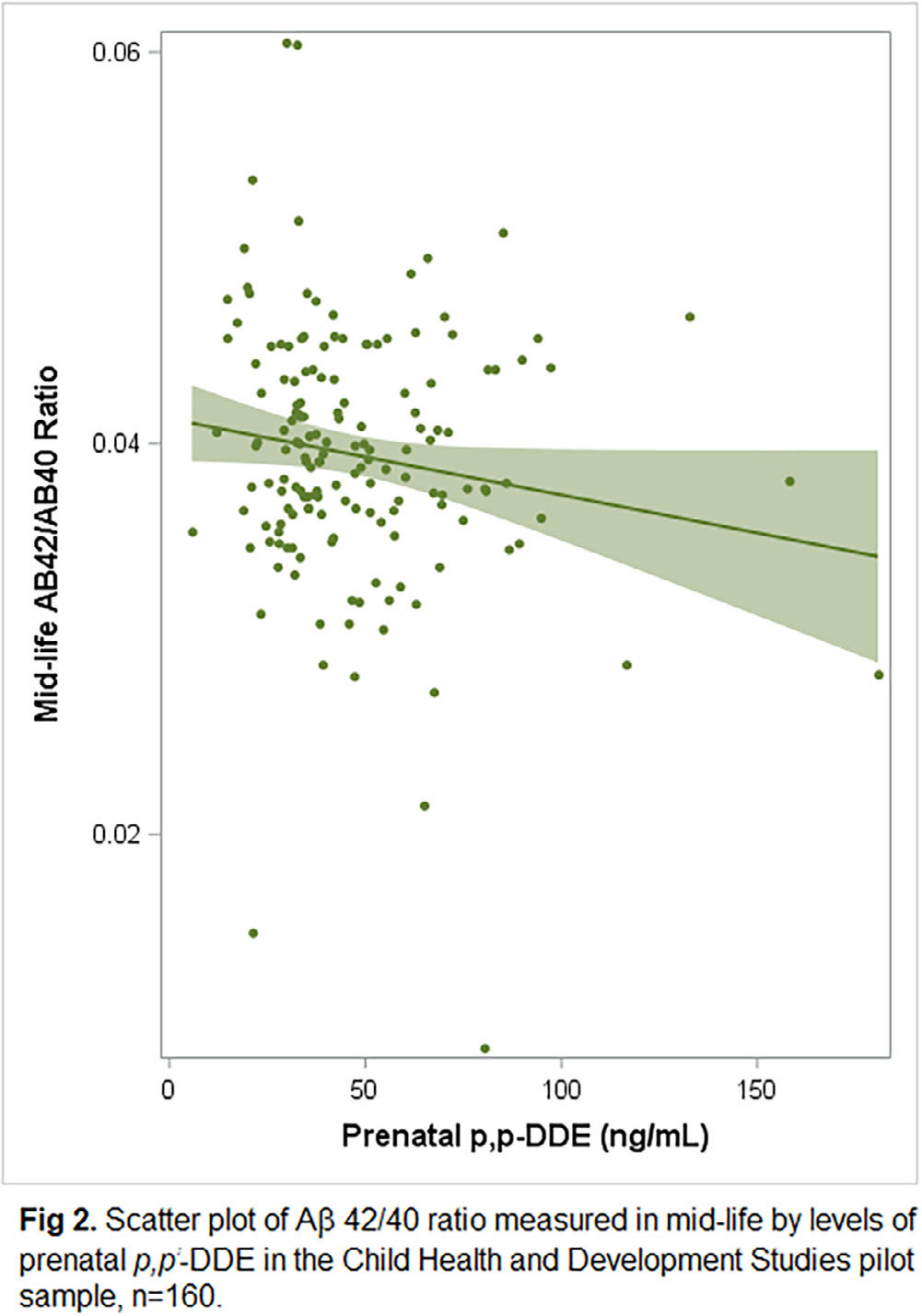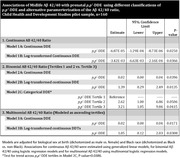# Association of Prenatal Pesticide Exposure with Plasma Aβ 42/40 Ratio in Midlife: Evidence from the Child Health and Development Studies

**DOI:** 10.1002/alz.092883

**Published:** 2025-01-09

**Authors:** Jason R Richardson, Isha Mhatre‐Winters, Piera Cirillo, Nickilou Krigbaum, Pam Factor‐Litvak, Young‐Mi Go, Dean P Jones, Barbara Cohn

**Affiliations:** ^1^ Isakson Center for Neurological Disease Research, College of Veterinary Medicine, University of Georgia, Athens, GA USA; ^2^ Child Health and Development Studies, Public Health Institute, Oakland, CA USA; ^3^ Columbia University, New York, NY USA; ^4^ Emory University, Atlanta, GA USA; ^5^ Department of Medicine, Emory University, Atlanta, GA USA

## Abstract

**Background:**

Environmental factors are increasingly being recognized as significant contributors to Alzheimer’s and Related Dementias (ADRD). Previously, we reported that higher serum concentrations of DDE, the highly persistent metabolite of the pesticide DDT was associated with increased risk of Alzheimer’s disease. We further demonstrated in cell and animal models that DDT and DDE increase amyloid pathology in the brain (Fig. 1). However, little is known about the effects of prenatal exposure on amyloid in humans.

**Method:**

Offspring born into the Child Health and Development Studies (CHDS) pregnancy cohort from 1960 ‐1963 in Oakland, CA were recruited for a diverse follow‐up study of health disparities in 2010. Participants completed cognitive function assessments, and provided interview data and blood samples, in their late 40’s to early 50’s. Aβ 42/40 was measured from the stored plasma taken in midlife using the Quanterix Neurology 3‐Plex A kit. Prenatal *p, p’*‐DDE, measured in archived maternal pregnancy serum, was available for this pilot sample, n = 160. Associations were estimated in linear, and binomial and multinomial logistic regression models, adjusted for race and sex.

**Result:**

Higher prenatal *p, p*‐DDE was associated with lower Aβ 42/40 in mid‐life (Fig. 2). In logistic models (Table 1), the Odds Ratio [OR] estimating lower Aβ 42/40 (tertiles 1 or 2 vs. tertile 3) = 2.6; 95% Confidence Limits [CL] = 1.0‐6.9 and OR = 3.2; 95% CL = 1.1‐9.8 for *p, p*‐DDE tertiles 2 and 3, respectively. Test of trend across *p, p’*‐DDE tertiles was significant (P‐value = 0.04). Associations were robust to multiple alternative modeling approaches (Table 1). Adjustment for achieved education in mid‐life did not alter results.

**Conclusion:**

Higher prenatal exposure to DDE is associated with lower plasma Aβ 42/40 in midlife, a putative prodromal marker for ADRD. Identification of individuals or populations more highly exposed to DDT/DDE may determine those at increased risk for ADRD later in life and allow for early intervention and prevention from progression to ADRD.